# MicroRNA alterations in sperm of infertile men: Insights into oligozoospermia, asthenozoospermia, and teratozoospermia

**DOI:** 10.1016/j.biotno.2025.10.004

**Published:** 2025-10-15

**Authors:** Manoharan Shunmuga Sundram, Sanjeeva Ready Nellapalli, Radha Vembu, Manjula Gopala Krishnan, Vettriselvi Venkatesan, Madhan Kalagara

**Affiliations:** a Department of Reproductive Medicine and Surgery, Sri Ramachandra Institute of Higher Education and Research (SRIHER), Porur, Chennai, 600 116, Tamil Nadu, India; b Department of Human Genetics, Sri Ramachandra Institute of Higher Education and Research (SRIHER), Porur, Chennai, 600 116, Tamil Nadu, India; c Vijaya medical centre, Visakhapatnam, 530 040, Andhra Pradesh, India

**Keywords:** Oligozoospermia, Teratozoospermia, Asthenozoospermia, male infertility, miRNA

## Abstract

**Background:**

Male infertility makes up almost 50 % of infertility cases in couples. Contributing factors include urogenital abnormalities, hormone imbalances, and genetic mutations. Current research highlights the important role of microRNAs (miRNAs) in male reproductive health, especially in regulating spermatogenesis. Altered expression of certain miRNAs have been linked to abnormal sperm issues like oligozoospermia (less number of sperm), asthenozoospermia (less sperm motility), and teratozoospermia(abnormal-shaped sperm). In this study, we looked at the potential of four candidate miRNAs, miR-139, miR-34b, miR-296, and miR-942, as biomarkers for male infertility.

**Methodology:**

Sperm samples were obtained from subjects subdivided into four groups based on seminal criteria including teratozoospermia, oligzoospermia, ashenozoospermia, and normospermia(healthy control). In the sperm samples, RNA was extracted using the TRIzol method. We measured miRNA expression using real-time PCR. The assessment of miR-139, miR-34b, miR-296,and miR-942 as diagnostic tools was analyzed using Receiver Operating Characteristic (ROC).

**Results:**

The investigation indicated elevated values of miR-139 in the asthenozoospermia, but miR-34b higher in teratozoospermia and miR-942 was observed to be high in all 3 groups, while miR-296 was lower in the three study groups. The motif characteristics of the ROC indicated good diagnostic capabilities for miR-139 and miR-942, as the AUC value was 0.8 (for patients with asthenozoospermia and oligozoospermia group).

**Conclusion:**

The results have shown that miR-139 in asthenozoospermia and miR-942 in oligozoospermia can be new candidate biomarkers for the diagnosis of male infertility. The use of miRNA in the diagnosis process can reveal several aspects of infertility and help to explore potential treatment in a more timely and targeted manner.

## 1 Introduction

Infertility is commonly considered to be the inability to conceive following 12 months or more of continuous, unprotected sexual activity. Infertility may also be defined as a reduction of oneself or a couple's ability to conceive.^1^ Male infertility is a rising concern in India. A study by the Indian Council of Medical Research shows that one in four couples faces infertility, with 10–15 % of cases attributed to male infertility.^2^ Evidences suggest that pure male factor infertility ranges from 2.5 % to 12 %, with rates varying by region: 4.5 %–6 % in North America, 9 % in Australia, and up to 12 % in Eastern Europe.^3^ In Nigeria, the rate is as high as 42.4 %.^4^ Globally, about one-sixth of couple's face infertility, with male factors contributing to half of these cases and being the sole cause in 20 %–30 % of cases.^5^ Average sperm counts have drastically decreased from 113 million/mL in 1940 to 66 million/mL in the 1990s, according to recent research, which is a worrying trend,^6^ and decreased by 51.6 % from 1973 to 2018.^7^ Male infertility can result from a range of factors including acquired urogenital abnormalities like blocked vas deferens, epididymitis, congenital issues e.g., undescended testes, ejaculatory duct cysts, endocrine disorders, environmental toxins like pesticides, smoking, genetic factors such as CFTR mutations, Klinefelter syndrome, and idiopathic causes. Other contributors include immunological conditions involving MicroRNA(miRNA) dysregulation, malignancies, medications, sexual dysfunction, and urogenital infections.^8^ Male infertility can occasionally go undiagnosed even after extensive clinical testing, including expensive and invasive treatments like testicular biopsies. The importance of miRNAs in controlling male fertility is becoming more widely recognized, and combining miRNA analysis with conventional diagnostic techniques may improve the precision of clinical diagnosis.^9^ Barbu et al., 2021^10^ demonstrated that miRNAs play a crucial role in determining various sperm characteristics, including sperm count, morphology, and motility. MiRNAs are present in all tissues and physiological fluids and play a role in both physiological and pathological processes. miRNA are abundant and stable in seminal liquid.^11^ It is essential for somatic cell development, oocyte fertilization, and spermatogenesis. Male infertility has been linked to abnormal levels of some miRNAs, which can impact sperm production and testicular function and result in diseases such azoospermia.^12^ This may result in problems with fertility, and infertile people with normal sperm parameters have been found to have abnormal miRNA expression profiles.^9^ miRNAs are small, endogenous, non-coding RNA molecules approximately 20–24 nucleotides long, that function as regulators of gene expression, and their dysregulation can profoundly affect male fertility, particularly in conditions like asthenozoospermia(decreased sperm motility), oligozoospermia (low sperm numbers) and teratozoospermia (abnormal sperm morphology).^13^ In oligozoospermia, miRNA dysregulation can affect spermatogenesis, leading to a reduced sperm count.^14^ In asthenozoospermia, miRNAs influence the motility of sperm by modulating the genes responsible for flagellar function and energy metabolism, which are essential for effective movement.^15^ In teratozoospermia, altered miRNA expression affects the proper development of sperm morphology, such as head and tail formation, which are critical for fertilization.^10^

In recent years, miRNAs have been extensively studied and recognized as promising biomarkers for various diseases due to their stability in body fluids, tissue-specific expression, and involvement in key pathophysiological processes.^16^^,^^17^ Consequently, miRNAs may serve as valuable markers for the diagnosis and treatment of male infertility and other reproductive disorders in men. This study aims to analyse the expression of four key miRNAs involved in spermatogenesis miR-139, miR-34b, miR-296, and miR-942 across different groups using real-time PCR. Furthermore, the diagnostic potential of these miRNAs will be evaluated through ROC (Receiver Operating Characteristic) analysis to assess their value as predictive biomarkers for male infertility.

## 2 Mechanism of miRNA in male infertility

Male infertility is a complex and multifactorial condition involving genetic, environmental, hormonal, and lifestyle factors. The miRNAs play an important role in the regulation of gene expression during spermatogenesis, the development of spermatozoa. The dysregulation of miRNAs can affect spermatogenesis and may lead to infertility, but it is only one aspect among many factors contributing to male infertility.^18^ Spermatogenesis begins with the mitotic division of spermatogonia, regulated by specific miRNAs for instance, miR-34b/c controls cell cycle checkpoints to ensure proper germ cell proliferation and prevent abnormalities like testicular cancer.^19^ The miR-139-5p has been implicated in male infertility by targeting genes in the activin signalling pathway, which inhibits *CYP26B1*, a critical regulator of male germ cell differentiation.^16^ miRNA like hsa-miR-296, which show differential expression between fertile and infertile men, highlight their potential role in the genetic basis of male infertility and suggest promising directions for future research and therapeutic strategies.^20^ The change from a proliferative to a differentiated state occurs when certain spermatogonia undergo differentiation into spermatocytes after proliferating. miRNAs like miR-21 promote this differentiation by targeting genes that inhibit the process, ensuring the steady progression of spermatogonia into spermatocytes.^21^ Spermatocytes go through a unique form of division during meiosis, which undergoes chromosome reduction to generate haploid spermatids.^22^ miRNAs function to regulate genes that are relevant to chromosomal pairing, recombination, and segregation. For example, miR-146 and miR-449 have been shown to regulate meiosis by targeting transcripts involved in chromatin remodelling and the synaptonemal complex, which are structures necessary for pairing chromosomes during meiosis.^23^ miRNAs can also prevent meiotic errors from taking place that can result in nondisjunction and aneuploidy - i.e. an abnormal number of chromosomes in sperm - and infertility.^24^ miR-221/222 clusters inhibit the expression of pro-apoptotic genes during meiosis, preventing cells from undergoing cell death during the meiotic process and allowing the meiotic process to progress normally.^25^ Furthermore, miR-296 has been found to promote meiotic processes in part by controlling expression of genes related to chromosome stability and meiotic spindle formation.^26^ During the final stages of spermatogenesis (spermatids), haploid spermatids undergo morphological & structural transformation to form mature spermatozoa.^27^ The transformation includes chromatin condensation, acrosome development, and flagellum formation.^27^ miRNAs are required for all stages of spermatid development. miR-10b regulates the development of proteins of vesicle trafficking and membrane fusion and consequently regulates acrosome formation, a cap-like structure. These features, namely chromatin condensation, acrosome differentiation, and flagellum development, are characterized by epigenetic phenomena, in which miRNAs are the key player for every aspect of this change. For instance, development and maturation of the acrosome depends on miR-10b′s role in regulating the levels of vesicle trafficking and membrane fusion proteins.^28^ In flagellum development, miR-135a targets proteins related to cytoskeletal organization, ensuring proper flagellum formation, which is essential for sperm motility.^29^ Additionally, miRNAs like miR-122 regulate the condensation of nuclear chromatin, which is crucial for protecting the sperm's genetic material during transit and fertilization by modulating the transition from histones to protamines, a key step in chromatin condensation.^30^ miR-942 is also involved in regulating the late stages of spermiogenesis, particularly influencing the structural changes necessary for sperm maturation and motility.^31^

**Fig. 1:**
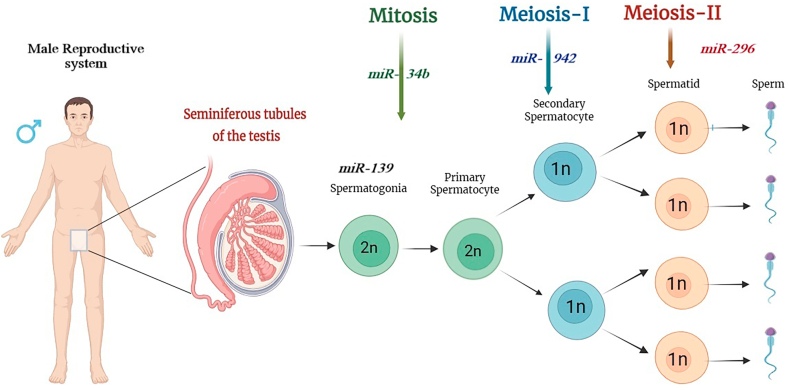
Key microRNAs Regulating Distinct Stages of Spermatogenesis.

miR-139 regulates spermatogenesis by maintaining spermatogonia stem cells and targeting genes involved in cell cycle regulation, apoptosis, and stress response, likely influencing the MAPK/ERK and PI3K/Akt pathways. miR-34b plays a key role in spermatogonial self-renewal, differentiation, and sperm morphology, targeting SIRT1 and the p53 pathway to regulate apoptosis and DNA damage response. miR-296 affects sperm motility and energy production by targeting mitochondrial function, ATP production, and cAMP signalling. miR-942 is linked to sperm motility and oxidative stress response, potentially modulating the NF-kB and PI3K/Akt pathways to influence sperm survival and function.^32^^,^^33^ In summary, miR-139, miR-34B, miR-296, and miR-942 are fundamental regulators of spermatogenesis, influencing processes such as germ cell proliferation, meiosis, and spermiogenesis illustrated in Fig. 1. Dysregulation of miRNAs can lead to various sperm abnormalities such as teratospermia [presence of sperm with abnormal morphology], asthenozoospermia [abnormal sperm motility], and oligozoospermia [low sperm count in the ejaculate], contribute to male infertility. Further research on specific miRNAs and their targets may offer new therapeutic approaches for treating male infertility.

## 3 Methodology

### 3.1 Sample collection and population parameters

The entire study was conducted at SRIHER. All patients provided written informed consent, and the Sri Ramachandra Institute of Higher Education and Research [SRIHER] Institutional Ethical Committee approved the study [IEC-NI/20/FEB/74/08].

Participants aged 28–35 years who adhered to a 3–5 days abstinence period and engaged in unprotected sexual activity for at least 12 months without achieving pregnancy were included. They were classified based on World Health Organization (WHO) 2021 seminal characteristics into teratozoospermia, asthenozoospermia, and oligozoospermia.^34^ Exclusion criteria included urogenital infections, systemic diseases, recent use of medications, reproductive surgeries, unhealthy lifestyle factors like smoking, alcohol, or drug use, improper sample collection, or non-compliance with abstinence. Individuals with prior identified genetic abnormalities (chromosomal disorders, Y-chromosome microdeletions) are also excluded. Normozoospermia (healthy control) participants were selected based on proven fertility, demonstrated by fathering a child within the past two years or achieving conception within 12 months of unprotected intercourse. Inclusion also required a semen analysis meeting WHO reference values for volume, concentration, motility, and morphology. Additionally, participants had no history of sexual or ejaculatory dysfunction, and their partners had no known female factor contributing to infertility. Individuals were excluded from the healthy control group if they had a history of smoking, use of recreational or illicit drugs, or occupational/environmental exposure to known reproductive toxicants. Other exclusion factors included any history of sexually transmitted infections (STIs), any genitourinary variables/history. In addition, participants who had fertility surgery (e.g., varicocelectomy, testicular biopsy, etc.), or were on hormonal or other medications determined to have the capability to change fertility, were also excluded. In total 88 sperm samples were collected from the Department of Reproductive Medicine and Surgery at Sri Ramachandra Medical Center and medical informed consent was obtained. The study groups consisted of (1) 20 individuals with teratozoospermia defined by abnormal sperm morphology, (2) 20 individuals with asthenozoospermia with poor sperm motility, (3) 20 individuals with oligozoospermia characterized by low sperm count, and (4) 28 individuals with normal clinically defined semen parameters, or normal, known as normozoospermia, where they were the healthy control group.

### 3.2 Sperm purification and RNA extraction

Semen samples were collected through masturbation into sterile, RNase-free containers. They were allowed to liquefy at room temperature for 30–60 min. After liquefaction, the semen was diluted with cold RNase-free phosphate-buffered saline (PBS) in a 1:1 ratio and centrifuged at 300×*g* for 10 min at 4 °C to pellet the cells. The supernatant was discarded, and the pellet was resuspended in 1 mL of cold Somatic Cell Lysis Buffer (0.1 % SDS and 0.5 % Triton X-100 in DEPC-treated water). The mixture was gently mixed by vortexing for 5–10 s and then incubated on ice for 10 min. This step selectively lysed contaminating round cells while keeping intact spermatozoa due to their strong membrane structure. The sample was centrifuged at 800×*g* for 10 min at 4 °C, and the resulting sperm pellet was washed twice with cold PBS to eliminate residual detergent.

RNA was extracted using the TRIzol method by adding 1 mL of TRIzol reagent to the sperm pellet. Vortexing ensured complete lysis, and the mixture was incubated at room temperature for 5 min. Chloroform (200 μL) was added, followed by vigorous shaking for 15 s. The mixture was then centrifuged at 12,000×*g* for 15 min at 4 °C to achieve phase separation. The RNA-containing aqueous phase was carefully transferred to a fresh tube. RNA was precipitated by adding an equal volume of isopropanol and centrifuging at 12,000×*g* for 10 min at 4 °C. The RNA pellet was air-dried for 20–30 min and then dissolved in RNase-free water, followed by incubation at 55–60 °C for 10–15 min to help complete dissolution.^35^ RNA quality and concentration were assessed using the Implen NanoPhotometer®. All samples showed concentrations of 40–50 ng/μL and A260/A280 purity values between 1.8 and 2.0.

### 3.3 miRNA cDNA conversion

The TaqMan Advanced miRNA cDNA Synthesis Kit was used to transform the chosen miRNAs into cDNA. The template was 1 μL of RNA. Five microliters were needed for the poly [A] tailing reaction, which included 2 μL of input RNA [concentrated at 10 ng/μL] and 3 μL of a master mix consisting of 10× Poly [A] Buffer, ATP, Poly [A] enzyme, and RNase-free water. This combination was incubated in a heat cycler for 55 min 5× DNA Ligase Buffer, 50 % PEG, 25× Ligation Adaptor, RNA Ligase, and RNase-free water were combined to create a reaction mix of 10 μL for adaptor ligation. After that, it was cycled at 16 °C for 60 min. By making the RT master mix, which contained 5× RT buffer, 25 mM dNTP mix, and 20× Universal RT primer, as instructed, using RNase-free water and a 10× RT enzyme mixture, reverse transcription was started immediately. After that, the reaction was cycled at 42 °C for 15 min. The products were ready for the miR-Amp reaction after reverse transcription. 2.5 μL of the RT product, 20 μL of the 2× miR-Amp master mix, 20× miR-Amp primer mix, and RNase-free water were added to form the final volume for miR-amplification. The mixture was then incubated for 45 min in a heat cycler. The cDNA samples were then diluted so that Real-Time PCR could measure miRNA expression. Four microRNAs and endogenous control were selected for this study based on previous associations with male infertility.^36^, ^37^, ^38^, ^39^ The expression of these microRNAs was assessed using pre-designed, commercially available TaqMan™ Advanced miRNA Assay Kits (Applied Biosystems, USA), which included miR-139-5p (Catalog No. 4427975), miR-34b-3p (Catalog No. 4427975), miR-942-5p (Catalog No. A25576), and miR-296-5p (Catalog No. 4427975). Quantification was achieved using the Rotor-Gene Q (Qiagen) and Applied Biosystems Fast 7900HT Real-Time PCR systems. miR-532-5p (Catalog No. A25576) was used as an endogenous control for expression normalization across samples.

### 3.4 Statistical evaluation

For every sample, the cycle threshold [Ct] values were noted for the target and control miRNA. Greater miRNA expression is indicated by lower Ct values. By comparing the target gene to the control gene, ΔCt values were computed, and ΔΔCt values were ascertained in relation to the average ΔCt of the control group. Formula 2^[−ΔΔCt] was used to determine each miRNA's relative expression. Each group's delta Ct values were analyzed separately in comparison to the control group. Statistical significance was determined using the student's t-test, with a p-value threshold of less than 0.05. The analysis was performed using the online tool Social Science Statistics. The sensitivity and specificity of the miRNAs as potential biomarkers were evaluated using Receiver Operating Characteristic [ROC] curves created with GraphPad Prism 8.0.2.

## 4 Result

### 4.1 Expression of miRNA with qRT-PCR

**Fig. 2:**
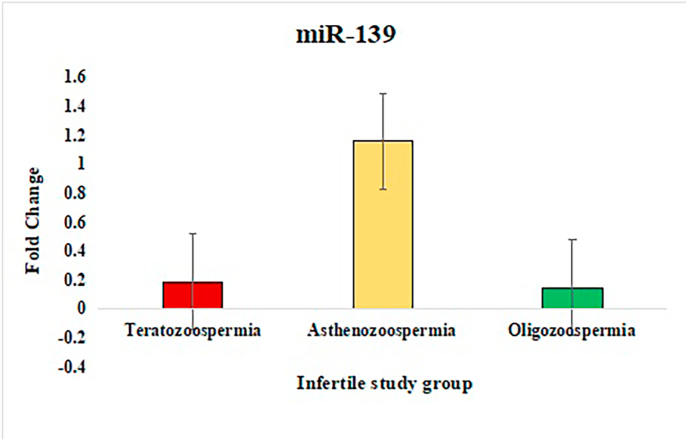
Relative fold change expression of miR-139 among the three infertile study groups.

**Fig. 3:**
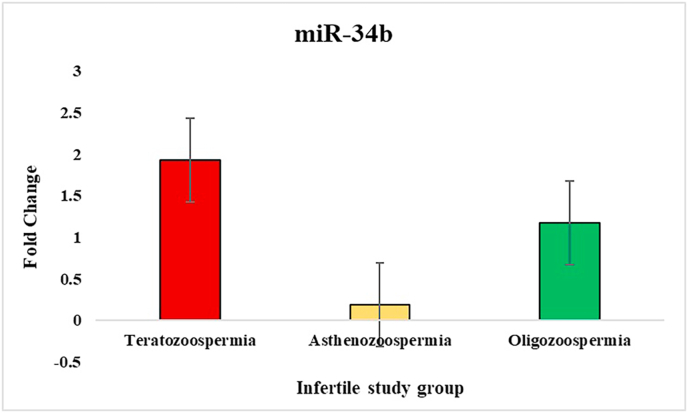
Relative fold change expression of miR-34b among the three infertile study groups.

**Fig. 4:**
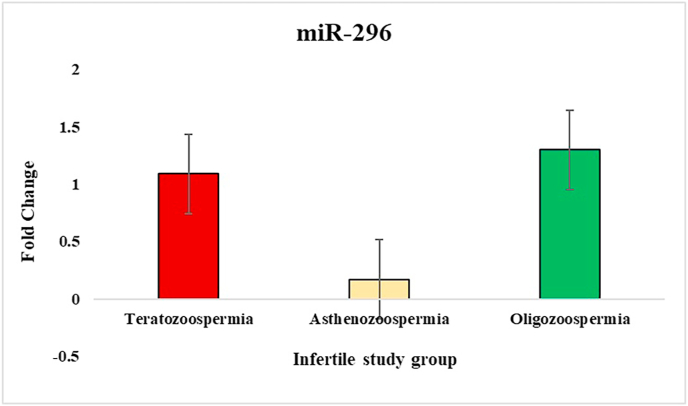
Relative fold change expression of miR-296 among the three infertile study groups.

**Fig. 5:**
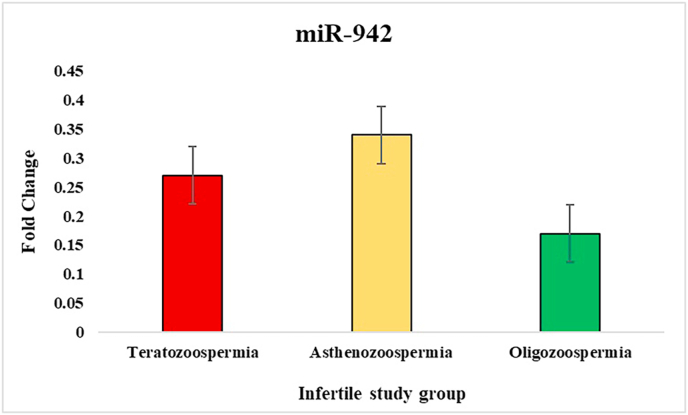
Relative fold change expression of miR-942 among the three infertile study groups.

In the study of sperm disorders, miRNA expression profiling revealed distinct patterns of dysregulation across key miRNAs involved in Teratozoospermia, Asthenozoospermia, and Oligozoospermia. miR-532-5p has been validated in the study that investigated miRNA profiles in semen, where it exhibited no significant alterations between fertile^40^ and infertile individuals, even in cases with abnormal seminal parameters like oligozoospermia or asthenozoospermia. This stability supports its role as a reliable internal control/endogenous. To normalize the results, miR-532-5p was employed as an endogenous control, ensuring accurate comparisons between patients and controls. Notably, in Asthenozoospermia, miR-139 exhibited a lower ΔCT value, indicating higher expression compared to the control group, with fold-change analysis confirming an upregulation by one-fold. This suggests that miR-139 might play a role in the mechanisms leading to reduced sperm count. miR-34b showed lower ΔCT values in teratozoospermic patients, indicating higher expression and implying its association with abnormal sperm morphology. The fold-change analysis also showed one-fold upregulation of miR-34b in teratozoospermic patients providing evidence of its possible role in the regulation of sperm morphology and development. In contrast miR-34b showed marked downregulation in the other two conditions, Oligozoospermia and Asthenozoospermia, where low expression of this miRNA may have significant implications for sperm function. miR-942 was upregulated across all three conditions, with Asthenozoospermia showing the highest fold change and statistical significance. This condition, characterized by reduced sperm motility, suggests a possible link between miR-942 and sperm motility dysfunction. For miR-296, higher ΔCT values were observed across all three patient groups compared to controls, indicating lower expression of miR-296 in these conditions. This consistent downregulation across Oligozoospermia, Teratozoospermia, and Asthenozoospermia hints at a broader role for miR-296 in the pathology of male infertility. Overall, these findings indicate that various miRNAs are distinctly expressed across different sperm disorders. The ΔCT values, fold-change (Fig. 2, Fig. 3, Fig. 4, Fig. 5) and p-value data were summarized in a detailed (Table 1), providing a clear comparison of miRNA expression levels between 3 different patient groups. This comprehensive presentation allows for easy identification of miRNAs that are upregulated or downregulated in each condition, highlighting their potential role in male infertility.

**Table:1:** Variation in ΔCT, Foldchange, and its p-value among the research groups.

miRNA	Teratozoospermia			Asthenozoospermia			Oligozoospermia		
	ΔCT	Foldchange	P-value	ΔCT	Foldchange	P-value	ΔCT	Foldchange	P-value
miRNA-139	0.59	0.19	0.24	−2.02	1.16	<0.0001∗	4.42	0.15	0.29
miRNA-34b	−1.66	1.93	0.18	0.61	0.19	0.85	−1.98	1.17	0.30
miRNA-942	1.02	0.27	0.19	0.7	0.34	0.04∗	1.67	0.17	0.09
miRNA-296	−0.91	1.09	0.16	1.68	0.17	0.25	−1.17	1.3	0.96

∗ Statistically significant.

### 4.2 Evaluation of Area under the curve (AUC) values as a potential biomarker

The diagnostic potential of four differentially expressed miRNAs was evaluated to assess their suitability as biomarkers for male infertility. When the sensitivity and specificity of each miRNA were plotted across several threshold values, a ROC curve was produced. The effectiveness of the biomarkers was measured by the AUC, where an AUC of 0.7–0.8 indicated a fair model, 0.8–0.9 represented a good model, and an AUC greater than 0.9 signified an excellent ability to differentiate between the two groups.^41^ In the teratozoospermic population, miR-942 showed moderate diagnostic value, with an AUC value of 0.7 and a significant p-value of 0.0163, indicating its relevance as a crucial biomarker. (Fig. 6a), indicating its effectiveness in distinguishing individuals within this group. In the asthenozoospermic group, miRNA-139 demonstrated even stronger diagnostic value, attaining a highly significant p-value of less than 0.0001 and an AUC of 0.8 (Fig. 6b), indicating its strong reliability in identifying asthenozoospermia cases. In the oligozoospermic group, miR-942 reaffirmed its role as a crucial biomarker, attaining an 0.8 AUC and a 0.0004 p-value (Fig. 6c), further emphasizing its importance in the detection of oligozoospermia. The AUC values for miR-34b and miR-296 are 0.6 and below imply in all the three study group. A low AUC suggests that while miRNA expression differences exist, they are not consistent or strong enough to be reliable diagnostic biomarkers. Factors like sample variability, biological complexity, and confounding variables may contribute to these low AUC values, limiting their diagnostic value. The combined analysis of all miRNAs yielded an AUC score of 0.64 (Fig. 6d). These findings highlight the potential utility of specific miRNAs as biomarkers for diagnosing various types of male infertility represented in (Table 2).

**Fig. 6a:**
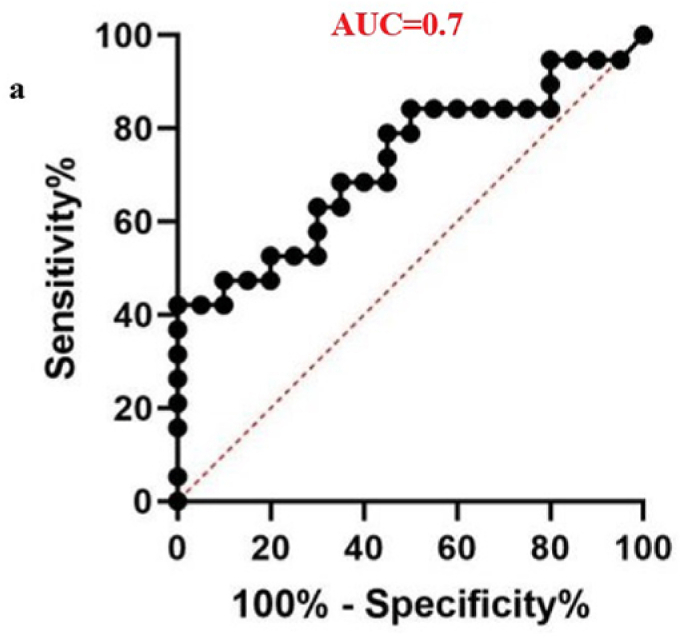
ROC analysis of miRNAs a miR-942 of teratozoospermic group.

**Fig. 6b:**
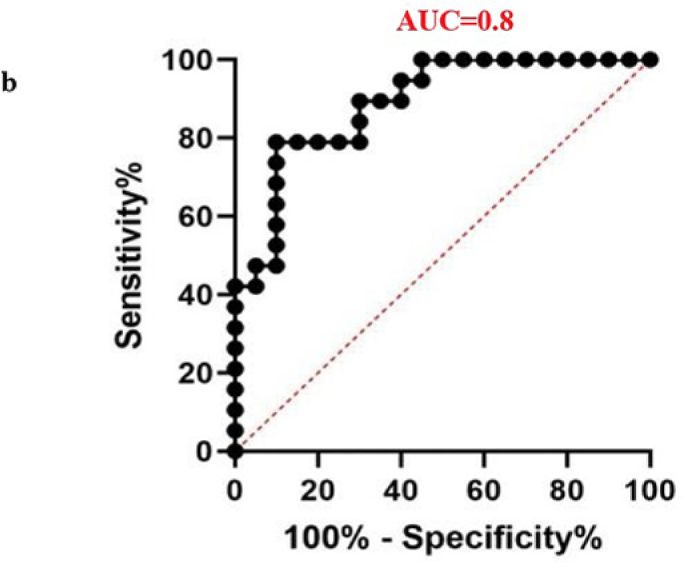
ROC analysis of miRNAs a miR-139 of asthenozoospermic group.

**Fig. 6c:**
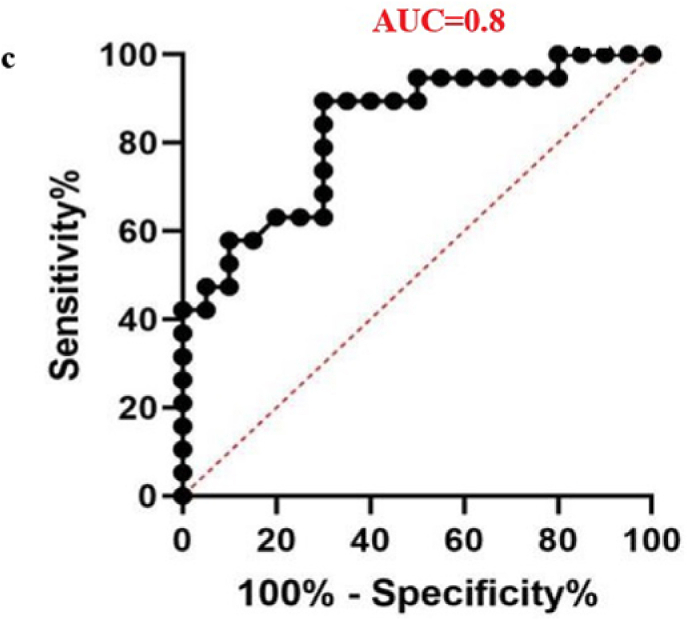
ROC analysis of miRNAs a miR-942 of oligozoospermia group.

**Fig. 6d:**
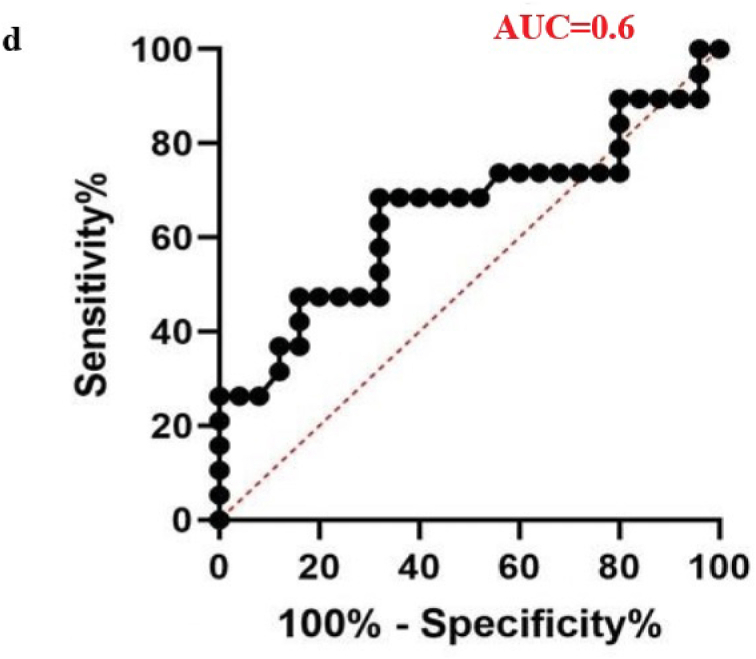
Combined ROC analysis of 4 miRNAs in 3 different study groups.

**Table 2:** AUC and P-Values in Infertile population.

miRNA	Teratozoospermia		Asthenozoospermia		Oligozoospermia	
	AUC	P-Value	AUC	P-Value	AUC	P-Value
miRNA-139	0.6645	0.0791	0.8895	<0.0001**∗**	0.5237	0.8004
miRNA-34b	0.5026	0.9776	0.5237	0.8004	0.5039	0.9664
miRNA-942	0.7250	0.0163∗	0.5395	0.6734	0.8303	0.0004**∗**
miRNA-296	0.6158	0.2163	0.6375	0.1779	0.5092	0.9217

∗ Statistically significant.

## 5 Discussion

Male infertility is still categorized as unexplained infertility in a considerable number of cases, despite a great deal of study being done to determine its causes. The histological evaluation of testicular tissue is one of the many standard procedures used today to diagnose unexplained male infertility, but it lacks the sensitivity and specificity necessary to provide a reliable diagnosis.^42^^,^^43^ Treatment options for male infertility in India include surgery, hormone therapy, assisted reproductive technologies (ART), and lifestyle changes. Surgery is often the first approach to address issues like obstructions or varicoceles in the reproductive tract. Hormone therapy may be recommended for men with low hormone levels essential for fertility.^44^

Some of the numerous cases of male infertility that were previously categorized as undetermined are believed to be caused by variations in the expression of specific miRNAs involved in the spermatogenesis process.^45^ However, recent studies have highlighted miRNAs as promising novel biomarkers that could help shed light on the underlying factors, offering new insights into the causes of male infertility and potentially resolving some of these uncertainties.^46^ Spermatogenesis involves around thousands of genes, and genetic changes in these genes, along with their regulators like miRNAs, which are emerging as important topics in infertility research.^47^ In a study, Tomic et al., found a considerable under-expressions of miR-296-5p in sperm from teratozoospermia patients, compared to healthy controls. In our study, we also found miR-296 was down regulated in all three infertile groups were down regulated which indicated a reduced expression levels in the sperm samples from patients with teratozoospermia.^48^ miR-34 family members, such as hsa-miR-449a, hsa-miR-34c-5p and hsa-miR-34b, predominantly expressed in the testis, were validated by Real time PCR and linked to cell proliferation, apoptosis, and differentiation.^49^ Similarly, our study showed that miR-34b had lower ΔCT values in teratozoospermic individuals, indicating increased expression in patients with abnormal sperm morphology. Fold-change analysis confirmed a 1-fold increase in miR-34b expression in teratozoospermia, suggesting a role in maintaining sperm structure. Corral-Vazquez et al., identified miR-942 showing the strongest potential for detecting seminal alterations being most effective in cases of unexplained infertility or borderline seminal parameters.^40^ Our study further supported the role of miR-942 in oligozoospermia, where its increased presence, indicated by a low ΔCT value and achieved an AUC of 0.8 and a p-value of 0.0004, reinforcing its diagnostic value for oligozoospermia as significance as a biomarker. It is believed that miR-139 is essential for controlling apoptosis in testicular cells, which is vital for ensuring the removal of defective germ cells and maintaining normal spermatogenesis. By promoting the clearance of abnormal cells, miR-139 contributes to the overall health of sperm production and development. This regulatory mechanism plays an important role in maintaining the integrity of the germ cell by excluding defective cells from spermatogenesis.^50^ In men with asthenozoospermia, miRNA-139 has demonstrated high sensitivity and specificity, thus achieving a high level of diagnostic threshold (AUC = 0.8) in testing. The diagnostic accuracy of the data is characterized by the value of p < 0.0001 for miRNA-139, which indicates a strong relationship that justifies that miRNA-139 has value as a reliable biomarker for identifying men with asthenozoospermia. Collectively these data suggest that miR-139 has an important functional role as a molecular regulator of healthy spermatogenesis, and in providing a diagnostic basis for some male fertility disorders such as asthenozoospermia.

## 6 Conclusion & future plans

miRNAs are important regulatory molecules for spermatogenesis. Abnormal expression levels have been found to be associated with cases of male infertility, including idiopathic infertility. Changes in expression levels of miR-139, miR-942, miR-34b, and miR-296 levels linked to specific sperm defects and profiling these miRNAs will help to understand the molecular basis for infertility. miR-139 and miR-942 provide the greatest diagnostic value. miRNA expression profiling will give some insight into unexplained infertility thereby providing new avenues for both diagnosis and possible treatments.

Future research will place greater emphasis on recruiting a larger and more ethnically diverse population into miRNA studies of male infertility, leading to stronger findings and generalizable knowledge between populations. A larger and more diverse cohort will allow researchers to determine whether the patterns of miRNA expression and the association to male infertility are global or not and whether there are other population-specific regulatory mechanisms. The research will include detailed functional studies of miRNAs related to male infertility, including but not limited to miR-139, miR-942, miR-34b, and miR-296. Functional studies will involve enough experiments to assess miRNA regulation of both spermatogenesis and sperm function, especially any analyses involving gene-knockdown models or even gene overexpressing models. Functional studies will provide more description to the target genes and biological pathways disrupted by miRNA regulation. This mechanistic work will also clarify the exact molecular functions of miRNAs associated with male infertility, and may inform the potential development of new therapies directed towards miRNAs.

## 7 Limitations

Although our findings are promising they are limited with our small number of participants. Repeating the study with larger with different ethnicities would strengthen the study. Larger heterogeneous samples would provide an opportunity to test if there are genetic or ethnic effects on miRNA expression and provide better information on the functional role of miRNA in male infertility. Establishing miRNAs specifically changed in infertility would give us stronger evidence for their potential clinical relevance.

## CRediT authorship contribution statement

**Manoharan Shunmuga Sundram:** Writing – original draft, Methodology, Formal analysis, Data curation, Conceptualization. **Sanjeeva Ready Nellapalli:** Writing – review & editing, Validation, Supervision. **Radha Vembu:** Writing – review & editing, Methodology, Investigation. **Manjula Gopala Krishnan:** Writing – review & editing, Validation, Conceptualization. **Vettriselvi Venkatesan:** Writing – review & editing, Visualization, Methodology, Investigation. **Madhan Kalagara:** Writing – review & editing, Validation, Software.

## Declaration of competing interest

The authors declare that they have no known competing financial interests or personal relationships that could have appeared to influence the work reported in this paper.
